
JOURNAL INFORMATION
==============================
NLM Title Abbreviation: South Med Rec
NLM Journal ID: 0027317

The Southern Medical Record

Publisher: Biomedical Journal Digitization Project

ARTICLE INFORMATION
==============================
PMCID: PMC9155534
PMID: 36025457
Article version: 1
Subjects: Prescription Department

Prescription Department

Print publication date: 1894 Nov
Volume: 24
Issue: 11
First page: 634
Last page: 635
License: This work is free of known copyright restrictions. For more information, please see PMC Back Issue Digitization.
License URL: https://creativecommons.org/publicdomain/mark/1.0/

==============================

TONSILLITIS.

Dr. Norbury recommends:

℞ Sulphide of lime, . . gr. iij.
Emul. poppy-seed, . ℥vj.
Syrup of opium, . . ℥j.
M. Sig. A tablespoonful every two hours.—*St. Louis Clinique.*

DIPSOMANIA.

By Dr. Edward C. Mann:

℞ Quininæ sulph., gr. ij.
Zinci oxid., gr. ij.
Strychninæ sulph., gr. 1–40.
Acid arseniosi, gr. 1–100.
Pulv. capsici, gr. ij.
M. et ft. pil. No. j. Sig. One pill thrice daily.

DIURETIC AND RHEUMATIC.

℞ Lithium salicylate....2 drams.
Liq. tong. sal ..........3 ozs.
Glycerine............1 oz.
Aqua mentha pip.......2 ozs.
M. Sig. Two teaspoonfuls every two hours.

FOR PYELITIS.

Dr. H. W. Moore, 724 South Twenty-second street, Philadelphia Pa., used with especially gratifying results in Pyelitis:

℞ "Sander & Sons" Eucalyptol.
Sig. Three drops three times a day.

In a case of an old sore leg, the following formula had been used with great benefit:

℞ Hydrarg. chlor. mit.. drams ijs.
Eucalyptol (Sander & Sons) drams ii.
Pulvis opii..............drams ijs.
Vaseline.... ........ounces ii.
M. Sig. Apply night and morning.

℞ Ammonii bromidi, ʒ iij.
Ammonii carbonat., gr. j.
Syrup tolu, f. ℥ j.
Aquæ dist. q. s. ad. f. ℥iij. M.
Sig. Teaspoonful every four hours.

FORMULA:
Each fluid drachm contains
Tonga, 30 grs.
Sodium Salicylate, 10 grs.
Ext. Cimicifugæ Racemosæ, 2 grs.
Pilocarpin Salicylate, 1-100 gr.
Colchicin Salicylate, 1-500 gr.

TONGALINE
ANTI-NEURALGIC.
ANTI-RHEUMATIC.
Possesses a peculiar affinity for viscid and sluggish secretions, neutralizing and eliminating them through the bowels and the emunctories.

INDICATED IN
Neuralgia,
Rheumatism,
La Grippe,
Gout,
Sciatica,
Nervous Headache.

The Salicylic Acid being from Oil of Wintergreen.

Sample of Tongaline sent to any physician who will pay express charges.

INDICATED IN
Metritis,
Endo-Metritis,
Subinvolution,
Menorrhagia,
Metrorrhagia,
Leucorrhœa,
Dysmenorrhœa,
Ovarian Neuralgia,
Painful Pregnancy,
After-Pains.

Ponca Comp.
UTERINE ALTERATIVE.
Exercises a decided alterative action on the uterine tissues, a general tonic influence on the Pelvic Organs; has a tendency to absorb plastic deposits, to regulate the vascular supply, to relieve congestion, to tone up the nerve forces, to encourage peristalsis, and to remove spasmodic conditions.

FORMULA:
Each tablet contains
Ext. Ponca, 3 grs.
Ext. Mitchella Repens 1 gr.
Caulophyllin, ¼ gr.
Helonin, ⅛ gr.
Viburnin, ⅛ gr.

Sample of Ponca Compound sent free on application.

MELLIER DRUG COMPANY, ST. LOUIS.

Pleas mention Southern Medical Record.

CORN CURE.

℞. Cocaine hydrochlorate....2 grs
Salicylic acid............30 grs
Alcohol.............120 minims
Solid ext. cannibis indica...8 grs
Collodion...........120 minims

Apply to the corn by any convenient means.

FOR BRONCHORRHEA.

℞. Copaiba...............drams iij
Tincturæ chloroform compositæ......................m xx
Liquoris potassæ ......drams j
Aquæ cinnamomi..ad ounces viij

Misce et fiat mistura.

An eighth part to be taken three times daily.—*Western Medical Reporter.*

BRONCHO PNEUMONIA.

For the cough:

℞. Antipyrin..............gr. viij
Syrup of cinchona.....ounces j
Syrup of eucalyptus....drams x
Peppermint water....ounces iss

M. Teaspoonful every hour.

If there is a tendency to collapse:

℞. Acetate of ammonium..drams ss
Malaga wine..........ounces ij
Syrup of ether........ drams v
Peppermint water.....drams x

M. Teaspoonful every hour.

During convalescence:

℞. Syrup of cinchona......dram x
Syrup of terpine.....ounces iss
Syrup of iodide of iron..ounces ij
Peppermint water.....ounces iss

M. A teaspoonful thrice daily.

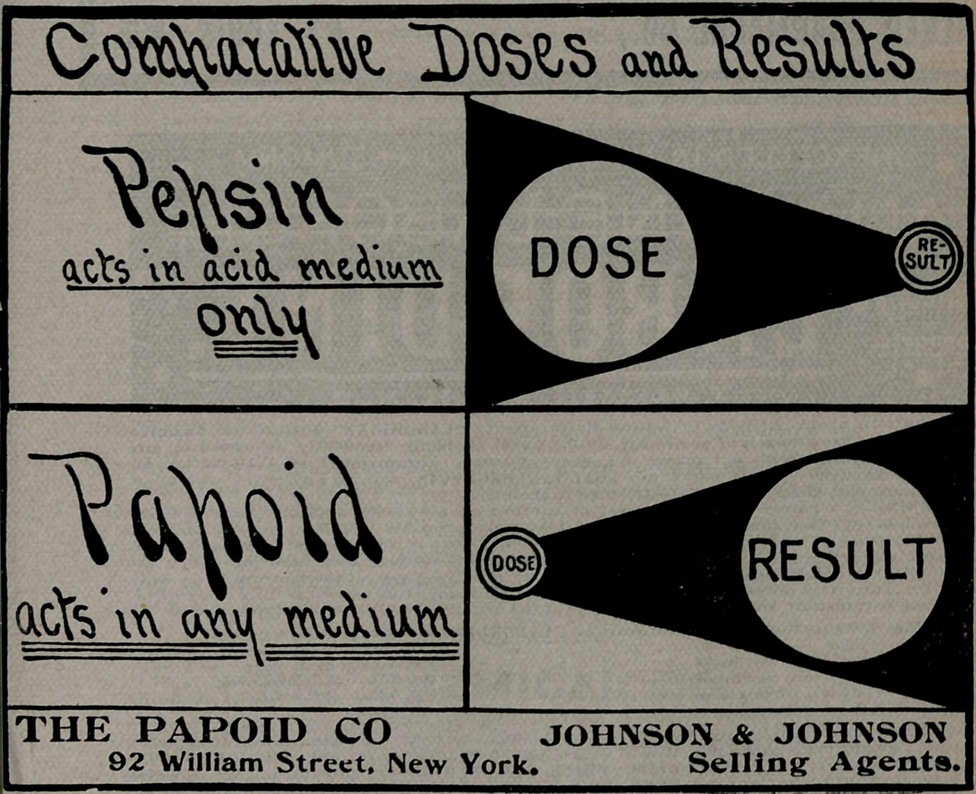

—*La Tribun Meep.*
